# Unprofessional conduct by nurses: A document analysis of disciplinary decisions

**DOI:** 10.1177/09697330211015289

**Published:** 2021-09-28

**Authors:** Oili Papinaho, Arja Häggman-Laitila, Mari Kangasniemi

**Affiliations:** University of Turku, Finland; Oulu University Hospital, Finland; University of Eastern Finland, Finland; Department of Social Services and Health Care, Finland; University of Turku, Finland

**Keywords:** Disciplinary action, document analysis, professional ethics, professional regulation, registered nurse, unprofessional conduct

## Abstract

**Background:**

A small minority of nurses are investigated when they fail to meet the required professional standards. Unprofessional conduct does not just affect the nurse but also patients, colleagues and managers. However, it has not been clearly defined.

**Objective:**

The objective was to identify unprofessional conduct by registered nurses by examining disciplinary decisions by a national regulator.

**Design:**

A retrospective document analysis.

**Data and research context:**

Disciplinary decisions delivered to 204 registered nurses by the Finnish national regulatory authority from 2007 to 2016. The data were analysed with quantitative statistics.

**Ethical consideration:**

The study received permission from the Finnish National Supervisory Authority for Welfare and Health and used confidential documents that were supplied on the basis of complete anonymity and confidentiality.

**Findings:**

The mean age of the registered nurses who were disciplined was 44 years and 81% were female. Two-thirds had worked for their employer for 5 years or less, 53% had two or more employers and 18% had a criminal history. All the decisions included a primary reason for why the nurses were investigated, but there were also 479 coexisting reasons. In most cases, unprofessional conduct was connected to substance abuse (96%). In addition, stealing of medicine, a decreased ability to work and neglect of nursing guidelines were reported.

**Discussion:**

We found that the nurses were investigated for unprofessional conduct for complex combinations of primary and coexisting reasons. Our study highlighted that more attention needs to be paid to the key markers for unprofessional conduct.

**Conclusion:**

Unprofessional conduct is a complex phenomenon that is connected to nurses’ individual and working backgrounds and has an impact on their work performance. More research is needed to identify how nursing communities can detect, manage and limit the serious effects and consequences of unprofessional conduct.

## Introduction

Nurses have a responsibility to ensure that they follow and observe professional ethics in their everyday work.^1,2^ However, most nurses have observed unprofessional conduct at some point during their working career.^3–9^ Unprofessional conduct^8,10,11^ refers to a nurse’s failure to meet the expected professional and ethical standards and legislation.^8,9,12–15^ It includes poor ethical competence and neglect of professional guidelines,^16,17^ not respecting patients’ rights and dignity and threatening patient safety.^7,18–20^ When nurses do not have knowledge, skills and abilities which they need to carry out their profession duties,^2,8^ this can lead to harmful incidents.^21^

When unprofessional conduct occurs, nurse managers and organisational administrators have the responsibility to intervene, monitor and resolve situations by using regulative protocols and issuing warnings or applying other sanctions.^22,23^ If the organisational procedures are insufficient, or there is a severe alleged breach of unprofessional conduct, national regulatory authorities are responsible for intervening and investigating cases.^22–24^ They must also evaluate the nurse’s ability to continue working and the legal consequences with regard to their professional rights.^22,25^ Based on previous research, nurses’ unprofessional conduct has been rarely studied at a national regulatory level.^26^ Regardless of the number of nurses a country has, the percentage of nurses that face disciplinary cases is quite similar. For example, in the United States, 0.2% of nurses from 1996 to 2006 were investigated at the highest level,^27^ in one Canadian province, less than 0.5% of nurses were disciplined from 2007 to 2017^28^ and the percentage was similar in Israel (0.24%) from 2002 to 2012.^18^ An Australian study reported that 175 disciplinary cases were investigated by the regulators from 1999 to 2006^7^ and in Brazil, 111 disciplinary cases from 2003 to 2013.^29^

In Finland, the number of investigated cases is in line with international levels, as each year, the regulatory authority investigates less than 0.3% of registered nurses who have seriously threatened patient safety.^30,31^ The licenced and regulated nursing degree is monitored by the board of the Finnish National Supervisory Authority for Welfare and Health, which is a part of the Ministry of Health and Social Affairs. The board also carries out investigations on nursing professionals when the authority has received allegations about unprofessional conduct from nurse managers, hospital administrators or other stakeholders. Organisations report these to the regulator if they feel that they are too serious to be dealt with at a local level and/or they might require sanctions that are not available to them. Other reasons are that the employer cannot guarantee patient safety because the sanctions that they are able to apply at a local level were not sufficient. Finally, the regulatory authority can instigate action itself if it becomes aware of issues relating to a particular nurse. The board of the regulatory authority consists of administrators and members who provide medical, legal and social care expertise. When they were considering disciplinary issues during the study period, they were joined by a member that represented the profession that was under investigation, such as a nursing professional when the complaint concerned a nurse. The board received written and oral reports of how patient safety had been seriously threatened, and it had the authority to sanction nurses if they neglected any of their professional obligations. These sanctions were temporary, permanent or indefinite and ranged from a warning or suspension to revocation of the nurse licence.^32^ In 2009–2018, the board issued decisions on about 40 cases of unprofessional conduct relating to registered nurses each year.^30^

Based on previous studies, nurses have been disciplined when they have committed errors in patients’ medication and documentation, and neglected to monitor patients or follow orders that have been given.^18,20,27,29,33–36^ Nurses have been disciplined for maltreating patients, practising without licence, carrying out tasks that exceed their professional remit and substance-related issues.^7,18,27,29,37–39^ Substance abuse has been frequently reported and has been shown to seriously affect nurses’ competence to practice. This was because it adversely affected their usual behaviour and attitudes and made them disregard instructions, increased their work absences, made it difficult for them to follow guidelines and increased the risk of accidents and near misses.^40–43^

Previous studies found that being over 40 years of age, male^20,27,34,35,44,45^ and having a criminal background^27,35,46^ were risk factors for disciplinary procedures. Other risks were a higher level nursing licence,^20,29,47^ working in long-term facilities or hospitals^18,29,34,35,48,49^ and prolonged contact with vulnerable patients.^37,50–52^ In addition, organisational and work environment factors increased the risks for unprofessional conduct. These included employers who were incapable of controlling ambiguous or hidden substance abuse. Other issues included lack of managerial abilities, high workload, lack of resources and working in haste or distractions during nursing tasks.^2,8,53,54^

A nurse’s unprofessional conduct does not just affect them; it also affects patient safety. In addition, nursing colleagues may be placed in a difficult situation if they are not sure whether to report suspicious incidents relating to a nurse.^5,6,8,55^ This can also require a higher level of moral courage,^17^ and those who report colleagues need adequate support.^56^ A nurse’s unprofessional conduct can also affect their work, the division of labour with their colleagues, their commitment to common professional rules and codes and the trust that society has in organisations and professionals.^56–58^ That is why greater knowledge of unprofessional conduct is needed, together with guidance on how to intervene when it occurs.

To protect the public, it is not enough to just discipline nurses who have failed to meet standards of practice. It is also important to understand unprofessional conduct so that future issues could be prevented and nurses who find themselves in difficult situations could be helped.

## Objective

The objective of this study was to identify unprofessional conduct by registered nurses by examining disciplinary decisions by a national regulator.

Our research questions (RQ) were as follows:

*RQ1*. What were the personal and working backgrounds of registered nurses who were disciplined for unprofessional conduct?
*RQ2*. What kind of unprofessional conduct was included in the disciplinary decisions issued by the Finnish regulatory authority following their investigations?
*RQ3*. How were the personal and working backgrounds of the nurses associated with unprofessional conduct?

## Method

### Study design

We conducted a retrospective document analysis^59^ to explore disciplinary decisions made by the Finnish regulatory authority against registered nurses. Disciplinary documents are important as they relate to whether justice is served. They provide systematically investigated data sources of unprofessional conduct in nursing, with unique and multi-perspective descriptions of the phenomenon.^60,61^

### Identifying the data

**Figure 1.**
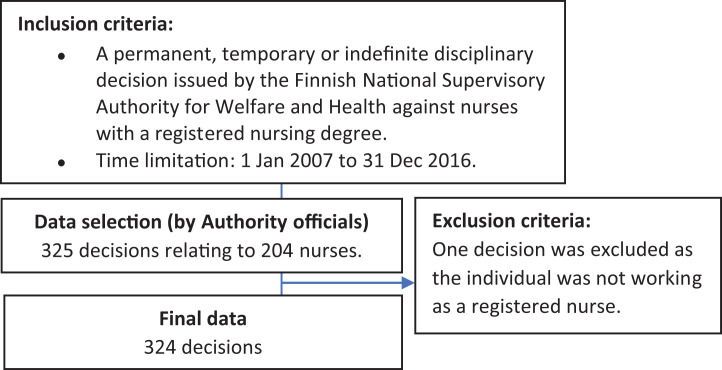
Data selection flow chart.

The research data consisted of disciplinary decisions against registered nurses made on unprofessional conduct cases by the board of the Finnish National Supervisory Authority for Welfare and Health. This study comprised data for the 10-year period from 1 January 2007 to 31 December 2016 (Figure 1).

### Data collection

We developed an electronic observation matrix that was based on the previous literature to collect the data.^7,18,29,41,62–65^ This comprised 34 open fields where we could record information about the nurses’ characteristics, when and why they were reported to the regulator, the facts of the case and the decisions that were issued. We tested the structure and the content of the observation matrix with the first 23 decisions that we collected. This testing process evaluated if the matrix included all the factors that we were able to explore by analysing the documents. Based on the inclusion criteria, the final data consisted of 324 decisions issued by the regulatory board on individual cases against 204 nurses.

The disciplinary decisions were written paper documents and consisted of the original report to the regulatory authority, the investigations and the decisions. Each decision consisted of approximately 50–450 pages of typed and/or hand-written text. The information for this study was collected from the decision documents and, in some cases, we extracted additional information from the attachments that led to the decisions. The information was manually transferred from the original paper documents to our electronic observation matrixes. We collected one observation matrix for each nurse and in some cases several decisions were combined into one observation matrix. According to the conditions that were laid down when permission was granted for the study, we collected the data in the regulatory authority’s office. This study analysed 13 of the 34 fields in the observation matrix. These covered the registered nurses’ personal and work background and when the report was submitted to the authority. The disciplinary decisions also stated the primary and coexisting reasons for why the nurses were investigated. We exported the content of the electronic observation matrix to numeric variables to analyse data.

### Data analysis

We used descriptive statistical methods and analysed them with a SPSS Statistics^®^, version 25.0 (IBM Corp, New York, USA). The categorical variables have been reported as frequencies and percentages for the variables and continuous variables as means and ranges for the year-based variables. We examined the statistical association between the registered nurses’ background and primary reasons as well as between the registered nurses’ background and criminal history. The Pearson correlation coefficient was used to calculate the statistical significance, which was set at *p* < 0.05. The results relate to the 204 registered nurses who were disciplined during the study period.

### Ethical considerations

This study followed the principles of good scientific practice. The study was carried out from October 2017 to March 2018 after we had received permission from the regulatory authority, which included clauses on the security and confidentiality of the data. The data that were collected contained information that had been collected by the authority for its investigation, not for research purposes.^60^ We only extracted data that were of direct relevance to our study, and the original documents were not printed, copied or scanned. The data were anonymised and saved as secured electronic forms.

## Results

### The personal and working backgrounds of the nurses who were disciplined

#### Personal background

The mean age of the 204 registered nurses was 44 years and 81% were female. All of them had a registered nursing degree and more than one-third had another degree (38%). In a quarter of cases, they had a lower educational level degree which had enabled them to work as a nurse assistant or licenced vocational nurse or emergency medical technician (25%). Another 15% had midwifery or public health nursing degrees. The mean time since they had graduated as a registered nurse was 16 years (Table 1). Almost one-fifth (18%) had an earlier criminal history.

**Table 1.** Personal and working backgrounds of the 204 nurses who were disciplined.

RN’s backgrounds	n	%	Mean	Range
Age				
years			43.5	25–61
25–34	39	19		
35–44	64	31		
45–54	75	37		
55–61	24	12		
Missing data	2	1		
Gender				
Female	166	81		
Male	38	19		
Nursing degree				
RN degree	127	62		
RN + low-level degree	47	23		
RN + same-level degree	26	13		
RN + same- and low-level degree	4	2		
Employment status				
RN	174	85		
Administrative position	17	8		
Other	7	4		
Missing data	9	3		
Working sector				
Public	155	76		
Private	43	21		
Other (third sector)	1	1		
Missing data	5	2		
Working organisation				
Hospital	106	52		
Supported healthcare facility and home healthcare	41	20		
Healthcare centre	26	13		
Other	14	7		
Missing data	17	8		
Clinical practice field				
Older people and geriatric care	41	20		
Medical, oncology and surgical care	30	15		
Accident, emergency and intensive care	29	14		
Community healthcare	25	12		
Mental and substance care	22	11		
Delivery, maternity and paediatric care	16	8		
Other	16	8		
Missing data	25	12		

RN: registered nurse.

#### Working background

Most of the registered nurses (85%) were working as nurses when they were reported for unprofessional conduct. Others held an administrative position as a junior charge nurse, a nursing manager or a nursing director (8%) or were working as an assistance nurse, a patient’s supervisor, a psychotherapist or an entrepreneur working in the social and healthcare sector (4%). Most of the registered nurses worked in the public sector (76%). More than half of the nurses worked in a hospital (52%), 20% worked in home healthcare roles, 13% worked in health centres and 7% in schools, occupational healthcare, social care and rehabilitation services (Table 1).

When it came to clinical practice fields, the nurses worked in older people and geriatric care (20%), medical, oncology or surgical care (15%), emergency or intensive care (14%) or community healthcare (12%). In addition, some nurses worked in mental and substance care (11%) and delivery, maternity and paediatric care (8%) (Table 1). They had worked for their current employer for an average of 5 years, and in 66% of cases, it was 5 years or less. Almost half of the registered nurses (48%) had two or more employers, more than quarter (28%) had just one employer and some of them (5%) were not permanently employed and worked sporadic shifts for organisations.

### Unprofessional conduct according to the disciplinary decisions

#### Primary reasons for unprofessional conduct

The primary reason for unprofessional conduct was reported for all 324 disciplinary decisions and most common was substance abuse (43%). This referred to nurses working, coming to work or spending time in the workplace under the influence of substances. In addition, substance use disorders or dependency was mentioned (18%). The second most frequent primary reason was stealing of medicine (32%). The third was the nurses’ reduced ability to work (14%), and it referred to substandard nursing competence and a low ability to work because of a health condition. The other reasons (10%) included falsifying documents, being suspected of a crime, not adhering to a prior regulatory agreement and stealing patients’ money (Table 2).

**Table 2.** Primary and coexisting reasons for unprofessional conduct.

Reasons	Primary reasons (n = 204)		Coexisting reasons (n = 479)			
	N	%	n	%	Mean	Range
Substance abuse	88	43.1	195	95.5	0.96	0–3
Working or coming to work under the influence of substances	50	24.5	84	41.2	0.41	0–1
Spending time in workplace under the influence of substances	*4*	*2.0*	22	10.8	0.11	0–1
Substance use disorder or dependence	38	18.6	89	43.6	0.44	0–1
Stealing medicine	66	32.4	93	45.6	0.46	0–1
Reduced ability to work	29	14.2	42	20.6	0.21	0–2
Substandard nursing competence	8	3.9	21	10.3	0.10	0–1
Low ability to work	21	10.3	21	10.3	0.10	0–1
Mental health condition	*12*	*5.9*	*16*	*3*	*0.08*	*0–1*
Memory problems	*4*	*2.0*	*5*	*2.5*	*0.02*	*0–1*
Other reasons	21	10.3	149	73.0	0.73	0–4
Falsifying documents	12	5.9	15	7.4	0.07	0–1
Suspected of a crime	5	2.5	7	3.4	0.03	0–2
Not adhering to a prior regulatory agreement	3	1.5	7	3.4	0.03	0–1
Stealing patients’ money	1	0.5				
Other reported coexisting reasons						
Unprofessional behaviour			38	18.6	0.19	0–1
Neglect of professional guidelines			78	38.2	0.38	0–3
Neglect of nursing practice			*28*	*13.7*	*0.14*	*0–1*
Neglect of the structures			*26*	*12.7*	*0.13*	*0–1*
Neglect of working time			*24*	*11.8*	*0.12*	*0–1*
Other threats to patient safety			4	2.0	0.02	0–1

#### The reported coexisting reasons for unprofessional conduct

The disciplinary decisions issued to the 204 registered nurses included the primary reasons and at least one other reason. These also related to unprofessional conduct, and 479 coexisting reasons were reported. The most frequently documented coexisting reason was substance abuse, as it was mentioned in the vast majority of cases (96%). The second was stealing of medicine, which was mentioned in almost half of the cases (46%). Just over a quarter (27%) of the nurses whose primary reason for unprofessional conduct was listed as substance abuse had stealing medicine recorded as a coexisting reason. Similarly, more than half of the nurses (52%) whose primary reason was listed as stealing medicine had substance abuse listed as a coexisting reason. That included nurses working, coming to work or spending time in the workplace under the influence of substances. The third frequent coexisting reason was that the nurse did not follow their professional guidelines (38%) including nursing practice, structures and working time. The other documented coexisting reasons were the nurses’ reduced ability to work (21%), unprofessional behaviour (19%) and other behaviour that threaten patient safety (2%) (Table 2).

### Association between the nurses’ backgrounds and unprofessional conduct

We found that registered nurses under the age of 44 years were more likely to steal medicine (43%) (*p* < 0.001) and that substance abuse was more common in those over 45 years (52%) (*p* = 0.017). No overall statistically significant association was found between gender and unprofessional conduct among the 166 female and 38 male registered nurses. The female nurses were more likely to commit substance abuse (45%), but apart from that, the patterns were fairly equal between the genders when it came to other forms of unprofessional conduct (Table 3).

**Table 3.** Association between RNs’ background and unprofessional conduct.

		Unprofessional conduct					
Background factors	n (%)	Substance abuse	*p* value	Stealing medicine	*p* value	Other unprofessional conduct	*p* value
	204 (100)	88 (43.1)		66 (32.6)		50 (24.5)	
Age (years)							
≤44	103 (50.5)	36 (35.0)	0.017**	44 (42.7)	0.001**	23 (22.3)	0.416
≥45	99 (48.5)	51 (51.5)		21 (21.2)		27 (27.3)	
Gender							
Female	166 (81.4)	75 (45.2)	0.218	53 (31.9)	0.786	38 (22.9)	0.261
Male	38 (18.6)	13 (34.2)		13 (34.2)		12 (31.6)	
Criminal history							
Yes	37 (18.1)	16 (43.2)	0.989	12 (32.4)	0.991	9 (24.3)	0.997
No	167 (81.9)	72 (43.1)		54 (32.3)		41 (24.6)	
RN degree							
RN only	153 (75.0)	68 (44.4)	0.514	48 (31.4)	0.604	37 (24.2)	0.851
RN with low-level degree	51 (25.0)	20 (39.2)		18 (35.3)		13 (25.5)	
Working career (years)							
<1	74 (36.2)	37 (50.0)	0.295	19 (25.7)	0.162	18 (24.3)	0.761
≥1	116 (56.9)	49 (42.2)		41 (35.3)		26 (22.4)	
Number of employers							
1	57 (27.9)	27 (47.4)	0.160	17 (29.8)	0.487	13 (22.8)	0.415
≥2	108 (52.9)	39 (36.1)		38 (35.2)		31 (28.7)	
Organisation							
Hospital	105 (51.5)	46 (43.8)	0.842	35 (33.3)	0.758	24 (22.9)	0.572
Another	99 (48.5)	42 (42.4)		31 (31.3)		26 (26.3)	

RN: registered nurse.

** Statistical significance, *p* < 0.05.

We found that the majority (88%) of the nurses with a criminal history were more likely (*p* = 0.002) to have two or more employers and more likely (*p* = 0.025) to have working contracts that had lasted less than a year (26%). The majority (79%) who worked for other organisations that were not hospitals had two or more employers (*p* = 0.001). In addition, 83% of the nurses who had a working contract of less than 1 year were more likely (*p* = 0.001) to have two or more employers (Table 4).

**Table 4.** Factors associated with registered nurses’ criminal backgrounds.

Backgrounds	n (%)	Employers		*p* value	Criminal history		*p* value
		1	≥2		Yes	No	
		57 (27.9)	108 (52.9)		37 (18.1)	167 (81.9)	
Working career (years)							
<1	74 (36.2)	12 (17.4)	57 (82.6)	<0.001**	19 (25.7)	55 (74.3)	0.025**
≥1	116 (56.9)	43 (48.3)	46 (51.7)		15 (12.9)	101 (87.1)	
Criminal history							
Yes	37 (18.1)	4 (11.8)	30 (88.2)	0.002**	–	–	–
No	(167 (81.9)	53 (40.5)	78 (59.5)		–	–	–
Organisation							
Hospital	105 (51.5)	40 (48.2)	43 (51.8)	<0.001**	17 (16.2)	88 (83.8)	0.457
Another	99 (48.5)	17 (20.7)	65 (79.3)		20 (20.2)	79 (79.8)	

** Statistical significance, *p* < 0.05.

## Discussion

This study showed that the reasons for why registered nurses were investigated for unprofessional conduct were a complex combination of primary and coexisting reasons. The main reason for unprofessional conduct was substance abuse, combined with neglecting professional ethics, reduced functional capacity and stealing medicines.^32^ When it came to the nurses’ personal and working backgrounds, unprofessional conduct was more common if they had a short working contract, several employers and a criminal history.

### Personal and working backgrounds of disciplined nurses

The disciplined nurses in our study differed from the general nursing population in Finland as they tended to be older and there were more males (19%).^31,66^ This male overrepresentation confirmed previous studies,^20,27,35,36,46,49^ but no statistical significance was found between females and males when we explored associations with unprofessional conduct. However, our results did demonstrate statistically significant differences between age groups with regard to unprofessional conduct, as it showed that nurses under 44 years were more likely to steal medicine and those over 45 years were more likely to be engaged in substance abuse.

Our results showed that registered nurses with a criminal history were overrepresented in disciplinary actions (18%) compared to the general nursing population. One study reported that 0.5% of all Finnish healthcare professionals have a criminal record.^67^ This overrepresentation was in line with previous studies.^27,35,46^ In future, more attention should be paid how to support disciplined nurses, regardless of their background, and how to support them with regard to their career. To do that, we need to investigate the factors that lead to unprofessional conduct.

In addition, our results demonstrated that, compared to previous studies, a higher number of nurses who were disciplined worked in the private sector.^31^ They were also more likely to work in home healthcare facilities or hospital settings than earlier studies.^44,49^ Although the mean age of the nurses who were disciplined was over 40 years, and they had graduated a mean of 16 years ago, they had a fragmented work history. They had typically been working for their current employer for 5 years, and nurses with contract of less than 1 year tended to have two or more employers. Shorter working contracts and numerous employers may demonstrate previous problems during a nurse’s career. Rapid turnover may be a way for a nurse to deny, and avoid, underlying problems, and this can make effective and appropriate managerial monitoring difficult.^8^ Employers can terminate the contracts of problematic nurses, rather than tackling their unprofessional conduct, but this just leaves them free to continue that behaviour in a future role. Nursing managers should have clear regulation codes and easy access to real-time information and the working backgrounds of nurses who apply to work with them. In addition, more attention needs to be paid to the root causes of unprofessional conduct by individual nurses, and employers need to be more aware of staff who have multiple employers or have changed jobs frequently. This would prevent the cumulation of individual problems and reduce the impact that unprofessional conduct can have on organisations, colleagues and patient safety.

### Complexity of unprofessional conduct

This study provides an overview of unprofessional nursing conduct that was reported in disciplinary decisions by a national regulatory authority. Being able to identify the primary and coexisting reasons can increase our understanding of the complexity of individual nurses’ multifaceted lives. The complexity of the phenomenon should make it easier to identify unprofessional conduct by individual nurses, as they will no doubt be displayed on the number of forms. In this study, the case included up to four coexisting reasons that explained why the nurses were investigated for unprofessional conduct. These combined multiple factors related to harmful incidents. However, previous studies have reported challenges when it comes to being aware of unprofessional conduct as a phenomenon and recognising it in clinical practice.^8,9,11^ In future, more attention need to be paid to the types of work communities where nurses’ unprofessional conduct has been identified. It needs to be clear what role nursing managers play in monitoring professional standards in clinical practice, together with the self-regulation role of the nursing profession. For example, different ethical support practices have improved awareness of ethical issues and made it easier for nursing managers to discuss ethical challenges.^68^ More research is also needed on what nurses should do when they become aware of colleagues who display unprofessional conduct.

Our finding that unprofessional conduct could result from a nurse’s reduced ability to work, such as substandard nursing competence, a lack of professional skill or health condition problems, confirms previous studies.^18,20^ Every nurse is responsible for their own professional competence and skills.^1^ However, organisations need to ensure that their staff provide a minimum level of safe, quality care.^54^ The need for nurses to take responsibility for their own professional and ethical competence cannot be overemphasised.

The nursing population is ageing, and this may explain why more are unable to work.^69^ We found that more than half of the registered nurses who were disciplined were over the age of 45 years. Healthcare professionals may have a higher risk of mental health problems and substance use disorders^11^ with associated personal problems.^8^ Substance abuse may also affect a nurse’s ability to work, and it needs to be seen as a disease that needs to be treated.^11,25,39^ In addition, health problems can also lead to substance abuse.^11^ Despite this, there has been very little research on how nurses’ professional, individual or contextual circumstances lead to unprofessional conduct at work.

Our study confirms research that found high rates of substance abuse among nurses who were disciplined.^25,43,64,70^ It also showed that substance abuse was the most frequent issue reported to the regulatory authority, as this poses serious risks for patient safety.^43^ Increased reports of the number of nurses abusing substances may demonstrate greater awareness of the issue, rather than more nurses using substances, as their rates are similar to the general population.^11,39,64,71^ In addition, higher reporting rates may be because substance abuse is easier to identify than other forms of unprofessional conduct in the workplace. It has been recognised that nurses face increased risk for substance abuse problems because their work is stressful and they have easy access to medication in their workplace and knowledge about how it can be used based on their profession. Stealing medicines often indicates substance abuse problems,^11^ which can be key markers for unprofessional conduct. Developing procedures to identify key signs of substance abuse could reduce the prevalence of this form of unprofessional conduct in the future. More systematic knowledge of the phenomenon is needed, not just for early prevention, but also for developing programmes to help nurses tackle substance abuse issues.

### Limitations

The limitations in this study were related to the research method and the data that were used. As the data in the documents, we reviewed, had not been produced for research purposes, some of the information we would have liked was missing. In addition, we did not report all the information that was available, such as the nurses’ country of origin or where they lived for ethical reasons. Even though we used total sampling, the number of the cases set a statistical limit when it came to analysing and exploring significant associations. We ensured the reliability of data by systematically developing an observation matrix. In addition, four researchers worked together to collect the data (OP, KL, MT, MK) and the data were double-checked by two researchers (OP, KL).

## Conclusion

Unprofessional conduct can cause serious consequences for the individual nurse, their organisation, managers, colleagues and patient safety. This review has demonstrated that unprofessional conduct is a complex phenomenon that involves multidimensional issues. Helping to identify the factors associated with unprofessional conduct can deepen our understanding of this harmful, but relatively rare, phenomenon, and improve the detection and management of problems at all regulatory levels. The most frequent cause of unprofessional conduct in our study was substance abuse, and our findings also provide important information on issues such as working background, contract length, age and criminal history. All cases of unprofessional conduct need to be considered as early as possible, and more research is needed to understand how to detect, manage and limit the serious affects and consequences of unprofessional conduct by registered nurses.
